# A Self-Adaptive Fuzzy* c*-Means Algorithm for Determining the Optimal Number of Clusters

**DOI:** 10.1155/2016/2647389

**Published:** 2016-11-29

**Authors:** Min Ren, Peiyu Liu, Zhihao Wang, Jing Yi

**Affiliations:** ^1^School of Information Science and Engineering, Shandong Normal University, Jinan, Shandong, China; ^2^School of Mathematic and Quantitative Economics, Shandong University of Finance and Economics, Jinan, Shandong, China; ^3^Shandong Provincial Key Laboratory for Distributed Computer Software Novel Technology, Jinan, Shandong, China

## Abstract

For the shortcoming of fuzzy* c*-means algorithm (FCM) needing to know the number of clusters in advance, this paper proposed a new self-adaptive method to determine the optimal number of clusters. Firstly, a density-based algorithm was put forward. The algorithm, according to the characteristics of the dataset, automatically determined the possible maximum number of clusters instead of using the empirical rule $\sqrt{n}$ and obtained the optimal initial cluster centroids, improving the limitation of FCM that randomly selected cluster centroids lead the convergence result to the local minimum. Secondly, this paper, by introducing a penalty function, proposed a new fuzzy clustering validity index based on fuzzy compactness and separation, which ensured that when the number of clusters verged on that of objects in the dataset, the value of clustering validity index did not monotonically decrease and was close to zero, so that the optimal number of clusters lost robustness and decision function. Then, based on these studies, a self-adaptive FCM algorithm was put forward to estimate the optimal number of clusters by the iterative trial-and-error process. At last, experiments were done on the UCI, KDD Cup 1999, and synthetic datasets, which showed that the method not only effectively determined the optimal number of clusters, but also reduced the iteration of FCM with the stable clustering result.

## 1. Introduction

Cluster analysis has a long research history. Due to its advantage of learning without a priori knowledge, it is widely applied in the fields of pattern recognition, image processing, web mining, spatiotemporal database application, business intelligence, and so forth.

Clustering is often as unsupervised learning and aims to partition objects in a dataset into several natural groupings, namely, the so-called clusters, such that objects within a cluster tend to be similar while objects belonging to different clusters are dissimilar. Generally, the datasets from different application fields vary in feature, and the purposes of clustering are multifarious. Therefore, the best method of cluster analysis depends on datasets and purposes of use. There is no universal clustering technology that can be widely applicable to the diverse structures presented by various datasets [1]. According to the accumulation rules of objects in clusters and the methods of applying these rules, clustering algorithms are divided into many types. However, for most clustering algorithms including partitional clustering and hierarchical clustering, the number of clusters is a parameter needing to be preset, to which the quality of clustering result is closely related. In practical application, it usually relies on users' experience or background knowledge in related fields. But in most cases, the number of clusters is unknown to users. If the number is assigned too large, it may result in more complicated clustering results which are difficult to be explained. On the contrary, if it is too small, a lot of valuable information in clustering result may be lost [2]. Thus, it is still a fundamental problem in the research of cluster analysis to determine the optimal number of clusters in a dataset [3].

Contributions of this paper are as follows. (1) A density-based algorithm is proposed, which can quickly and succinctly generate high-quality initial cluster centroids instead of choosing randomly, so as to stabilize the clustering result and also quicken the convergence of the clustering algorithm. Besides, this algorithm can automatically estimate the maximum number of clusters based on the features of a dataset, hereby determining the search range for estimating the optimal number of clusters and effectively reducing the iterations of the clustering algorithm. (2) Based on the features of compactness within a cluster and separation between clusters, a new fuzzy clustering validity index (CVI) is defined in this paper avoiding its value close to zero along with the number of clusters tending to the number of objects and obtaining the optimal clustering result. (3) A self-adaptive method that iteratively uses improved FCM algorithm to estimate the optimal number of clusters was put forward.

## 2. Related Work

The easiest method of determining the number of clusters is data visualization. For the dataset that can be effectively mapped to a 2-dimensional Euclidean space, the number of clusters can be intuitively acquired through the distribution graph of data points. However, for high-dimensional and complicated data, this method is unserviceable. Rodriguez and Laio proposed a clustering algorithm based on density peaks, declaring that it was able to detect nonspherical clusters and to automatically find the true number of clusters [4]. But, in fact, the number of cluster centroids still needs to be selected artificially according to the decision graph. Next, relevant technologies to determine the optimal number of clusters are summarized below.

### 2.1. Clustering Validity Index Based Method

Clustering validity index is used to evaluate the quality of partitions on a dataset generated by the clustering algorithm. It is an effective method to construct an appropriate clustering validity index to determine the number of clusters. The idea is to assign different values of the number of clusters *c* within a certain range, then run fuzzy clustering algorithm on the dataset, and finally to evaluate the results by clustering validity index. When the value of clustering validity index is of the maximum or the minimum or an obvious inflection point appears, the corresponding value of *c* is the optimal number of clusters *c*_opt_. So far, researchers have put forward lots of fuzzy clustering validity indices, divided into the following two types.

*(1) Clustering Validity Index Based on Fuzzy Partition*. These indices are according to such a point of view that for a well-separated dataset, the smaller the fuzziness of fuzzy partition is, the more reliable the clustering result is. Based on it, Zadeh, the founder of fuzzy sets, put forward the first clustering validity index degree of separation in 1965 [5]. But its discriminating effect is not ideal. In 1974, Bezdek put forward the concept of partition coefficient (PC) [6] which was the first practical generic function for measuring the validity of the fuzzy clustering and subsequently proposed another clustering validity function partition entropy (PE) [7] closely related to partition coefficient. Later, Windham defined proportion exponent by use of the maximum value of a fuzzy membership function [8]. Lee put forward a fuzzy clustering validity index using the distinguishableness of clusters measured by the object proximities [9]. Based on the Shannon entropy and fuzzy variation theory, Zhang and Jiang put forward a new fuzzy clustering validity index taking account of the geometry structure of the dataset [10]. Saha et al. put forward an algorithm based on differential evolution for automatic cluster detection, which well evaluated the validity of the clustering result [11]. Yue et al. partitioned the original data space into a grid-based structure and proposed a cluster separation measure based on grid distances [12]. The clustering validity index based on fuzzy partition is only related to the fuzzy degree of membership and has the advantages of simpleness and small calculating amount. But it lacks direct relation with some structural features of the dataset.

*(2) Clustering Validity Index Based on the Geometry Structure of the Dataset*. These indices are based on such a point of view that for a well-separated dataset, every cluster should be compact and separated from each other as far as possible. The ratio of compactness and separation is used as the standard of clustering validity. This type of representative clustering validity indices include Xie-Beni index [13], Bensaid index [14], and Kwon index [15]. Sun et al. proposed a new validity index based on a linear combination of compactness and separation and inspired by Rezaee's validity [16]. Li and Yu defined new compactness and separation and put forward a new fuzzy clustering validity function [17]. Based on fuzzy granulation-degranulation, Saha and Bandyopadhyay put forward a fuzzy clustering validity function [18]. Zhang et al. adopted Pearson correlation to measure the distance and put forward a validity function [19]. Kim et al. proposed a clustering validity index for GK algorithm based on the average value of the relative degrees of sharing of all possible pairs of fuzzy clusters [20]. Rezaee proposed a new validity index for GK algorithm to overcome the shortcomings of Kim's index [21]. Zhang et al. proposed a novel WGLI to detect the optimal cluster number, using global optimum membership as the global property and modularity of bipartite network as the local independent property [22]. The clustering validity index based on the geometric structure of the dataset considers both the fuzzy degree of membership and the geometric structure, but its membership function is quite complicated with large calculating amount.

Based on clustering validity index, the optimal number of clusters is determined through exhaustive search. In order to increase the efficiency of estimating the optimal number of clusters *c*_opt_, the search range of *c*_opt_ must be set; that is, *c*_max⁡_ is the maximum number of clusters, assigned to meet the condition *c*_opt_ ≤ *c*_max⁡_. Most researchers used the empirical rule $c_{\max}\leq \sqrt{n}$, where *n* is the number of data in the dataset. For this problem, theoretical analysis and example verification were conducted in [23], which indicated that it was reasonable in a sense. However, apparently, this method has the following disadvantages. (1) Each *c* must be tried in turn, which will cause a huge calculation. (2) For each *c*, it cannot be guaranteed that the clustering result is the globally optimal solution. (3) When noise and outliers are existing, the reliability of clustering validity index is weak. (4) For some datasets like FaceImage [24], if $c\geq \sqrt{n}$, the empirical rule will be invalid. Researches showed that due to the diversified data types and structures, no universal fuzzy clustering validity index can be applicable to all datasets. The research is and will still be carried on urgently.

### 2.2. Heuristic Method

Some new clustering algorithms have been proposed in succession recently. The main idea is to use some criteria to guide the clustering process, with the number of clusters being adjusted. In this way, while the clustering is completed, the appropriate number of clusters can be obtained as well. For example, *X*-means algorithm [25] based on the split hierarchical clustering is representative. Contrary to the aforesaid process, RCA [26] determined the actual number of clusters by a process of competitive agglomeration. Combining the single-point iterative technology with hierarchical clustering, a similarity-based clustering method (SCM) [27] was proposed. A mercer kernel-based clustering [28] estimated the number of clusters by the eigenvectors of a kernel matrix. A clustering algorithm based on maximal *θ*-distant subtrees [29] detected any number of well-separated clusters with any shapes. In addition, Frey and Dueck put forward an affinity propagation clustering algorithm (AP) [24] which generated high-quality cluster centroids via the message passing between objects to determine the optimal number of clusters of large-scale data. Shihong et al. proposed a Gerschgorin disk estimation-based criterion to estimate the true number of clusters [30]. José-García and Gómez-Flores presented an up-to-date review of all major nature-inspired metaheuristic algorithms used thus far for automatic clustering, determining the best estimate of the number of clusters [31]. All these have widened the thoughts for relevant researches.

## 3. Traditional FCM for Determining the Optimal Number of Clusters

Since Ruspini first introduced the theory of fuzzy sets into cluster analysis in 1973 [32], different fuzzy clustering algorithms have been widely discussed, developed, and applied in various areas. FCM [33] is one of the most commonly used algorithms. FCM was first presented by Dunn in 1974. Subsequently, Bezdek introduced weighted index to the fuzzy degree of membership [34], further developing FCM. FCM divides the dataset *X* into *c* fuzzy clusters. This algorithm holds that each object belongs to a certain cluster with a different degree of membership, that is, a cluster is considered as a fuzzy subset on the dataset.

Assume that *X* = {*x*_1_, *x*_2_,…, *x*_*n*_} is a finite dataset, where *x*_*i*_ = (*x*_*i*1_, *x*_*i*2_,…, *x*_*il*_) is an l-dimensional object and *x*_*ij*_ is the *j*th property of the *i*th object. *C* = {*C*_1_, *C*_2_,…, *C*_*c*_} denotes *c* cluster(s). *V* = {*v*_1_, *v*_2_,…, *v*_*c*_} represents *c* l-dimensional cluster centroid(s), where *v*_*i*_ = (*v*_*i*1_, *v*_*i*2_,…, *v*_*il*_). *U* = (*u*_*ik*_)_(*n*×*c*)_ is a fuzzy partition matrix, and *u*_*ik*_ is the degree of membership of the *i*th object in the *k*th cluster, where ∑_*k*=1_^*c*^ *u*_*ik*_ = 1, ∀*i* = 1,…, *n*. The objective function is the quadratic sum of weighed distances from the samples to the cluster centroid in each cluster; that is,

(1) $$\begin{array}{c} J_{m}\left(\;U,V\;\right)=\overset{c}{\underset{k=1}{\sum }}\overset{n}{\underset{i=1}{\sum }}u_{ik}^{m}d_{ik}^{2}, \end{array}$$

where *d*_*ik*_ = ‖*x*_*i*_ − *v*_*k*_‖ shows the Euclidean distance between the *i*th object and the *k*th cluster centroid. *m* (*m* ∈ [1, *∞*)) is a fuzziness index, controlling the fuzziness of the memberships. As the value of *m* becomes progressively higher, the resulting memberships become fuzzier [35]. Pal and Bezdek advised that *m* should be between 1.5 and 2.5, and usually let *m* = 2 if without any special requirement [36].

According to the clustering criterion, appropriate fuzzy partition matrix *U* and cluster centroid *V* are obtained to minimize the objective function *J*_*m*_. Based on the Lagrange multiplier method, *U* and *V* are, respectively, calculated by the formulas below:

(2) uik=1∑j=1cdik/djk2/m−1,

(3) vk=∑i=1nuikmxi∑i=1nuikm.

FCM algorithm is carried out through an iterative process of minimizing the objective function *J*_*m*_, with the update of *U* and *V*. The specific steps are as follows.

**Step 1 .**

Assign the initial value of the number of clusters *c*, fuzziness index *m*, maximum iterations *I*_max⁡_, and threshold *ξ*.

**Step 2 .**

Initialize the fuzzy partition *U* ^(0)^ randomly according to the constraints of the degree of membership.

**Step 3 .**

At the *t*-step, calculate *c* cluster centroids *V* ^(*t*)^ according to (3).

**Step 4 .**

Calculate the objective function *J*_*m*_^(*t*)^ according to (1). If |*J*_*m*_^(*t*)^ − *J*_*m*_^(*t* − 1)^| < *ξ* or *t* > *I*_max⁡_, then stop; otherwise continue to Step  5.

**Step 5 .**

Calculate *U* ^(*t*+1)^ according to (2) and return to Step  3.

At last, each object can be arranged into one cluster in accordance with the principle of the maximum degree of membership. The advantages of FCM may be summarized as simple algorithm, quick convergence, and easiness to be extended. Its disadvantages lie in the selection of the initial cluster centroids, the sensitivity to noise and outliers, and the setting of the number of clusters, which have a great impact on the clustering result. As the random selection of the initial cluster centroids cannot ensure the fact that FCM converges to an optimal solution, different initial cluster centroids are used for multiple running of FCM; otherwise they are determined by using of other fast algorithms.

The traditional method to determine the optimal number of clusters of FCM is to set the search range of the number of clusters, run FCM to generate clustering results of different number of clusters, select an appropriate clustering validity index to evaluate clustering results, and finally obtain the optimal number of clusters according to the evaluation result. The method is composed of the following steps.

**Step 1 .**

Input the search range [*c*_min⁡_, *c*_max⁡_]; generally, *c*_min⁡_ = 2 and $c_{\max}=\lfloor \sqrt{n}\rfloor$.

**Step 2 .**

For each integer *kn* ∈ [*c*_min⁡_, *c*_max⁡_].

*Step  2.1.* Run FCM.

*Step  2.2.* Calculate clustering validity index.

**Step 3 .**

Compare all values of clustering validity index. *kn* corresponding to the maximum or minimum value is the optimal number of clusters *c*_opt_.

**Step 4 .**

Output *c*_opt_, the optimal value of clustering validity index, and clustering result.

## 4. The Proposed Methods

### 4.1. Density-Based Algorithm

Considering the large influence of the randomly selected initial cluster centroid on the clustering result, a density-based algorithm is proposed to select the initial cluster centroids, and the maximum number of clusters can be estimated at the same time. Some related terms will be defined at first.

**Definition 1 (local density).**

The local density *ρ*_*i*_ of object *x*_*i*_ is defined as

(4) $$\begin{array}{c} \rho_{i}=\overset{n}{\underset{j=1}{\sum }}e^{-d_{ij}^{2}/dc^{2}}, \end{array}$$

where *d*_*ij*_ is the distance between two objects *x*_*i*_ and *x*_*j*_ and *dc* is a cutoff distance. The recommended approach is to sort the distance between any two objects in descending order and then assign *dc* as the value corresponding to the first *p*% of the sorted distance (appropriately *p* ∈ [2,5]). It shows that the more objects with a distance from *x*_*i*_ less than *dc* there are, the bigger the value of *ρ*_*i*_ is. The cutoff distance finally can decide the number of initial cluster centroids, namely, the maximum number of clusters. The density-based algorithm is robust with respect to the choice of *dc*.

**Definition 2 (core point).**

Assume that there is an object *x*_*i*_ ∈ *X*, if *x*_*i*_ ∉ *C*_*k*_ and *ρ*_*i*_ = max_*x*_*j*_∉*C*_*k*__⁡(*ρ*_*j*_), *k* = 1,…, *c*, *j* = 1,…, *n*, then *x*_*i*_ is a core point.

**Definition 3 (directly density-reachable).**

Assume that there are two objects *x*_*i*_, *x*_*j*_ ∈ *X*, if their distance *d*_*ij*_ < *dc*, then *x*_*j*_ is directly density-reachable to *x*_*i*_ and vice versa.

**Definition 4 (density-reachable).**

Assume that *X* is a dataset and objects *x*_*s*_, *x*_*s*+1_,…, *x*_*t*−1_, *x*_*t*_ belong to it. If, for each *i* ∈ [*s*, *t* − 1], *x*_*i*+1_ is directly density-reachable to *x*_*i*_, then *x*_*s*_ is density-reachable to *x*_*t*_.

**Definition 5 (neighbor).**

Neighbors of an object *x*_*i*_ are those who are directly density-reachable or density-reachable to it, denoted as Neighbor (*x*_*i*_).

**Definition 6 (border point).**

An object is called a border point if it has no neighbors.

**Figure 1:**
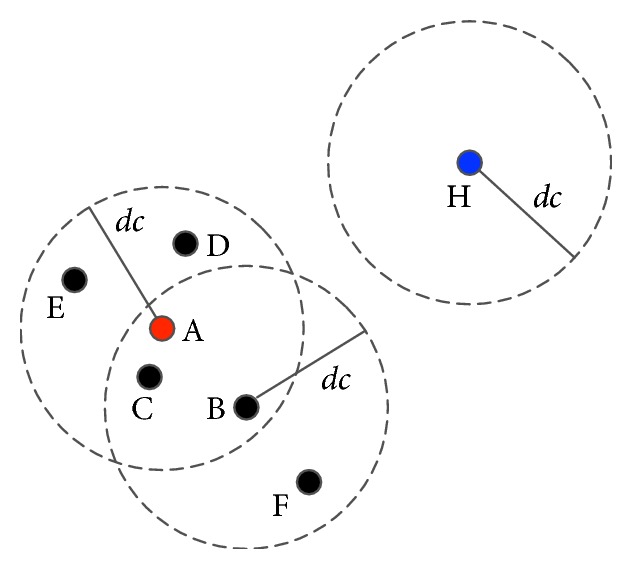
An example. Point A is of the highest local density. If A does not belong to any cluster, then A is a core point. Points B, C, D, and E are directly density-reachable to point A. Point F is density-reachable to point A. Point H is a border point.

The example is shown in Figure 1.

**Figure 2:**
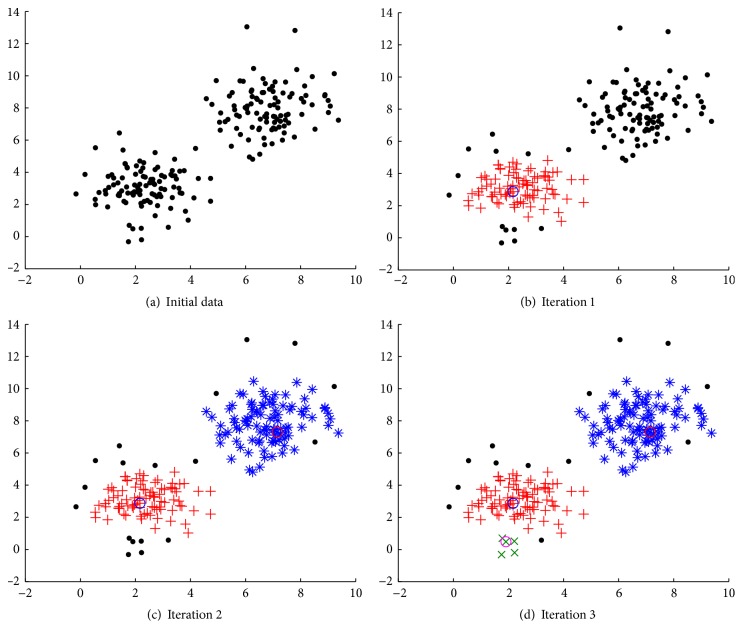
Demonstration of the process of density-based algorithm. (a) is the initial data distribution of a synthetic dataset. The dataset consists of two pieces of 2-dimensional Gaussian distribution data with centroids, respectively, as (2, 3) and (7, 8). Each class has 100 samples. In (b), the blue circle represents the highest density core point as the centroid of the first cluster, and the red plus sign represents the object belonging to the first cluster. In (c), the red circle represents the core point as the centroid of the second cluster, and the blue asterisk represents the object belonging to the second cluster. In (d), the purple circle represents the core point as the centroid of the third cluster, the green times sign represents the object belonging to the third cluster, and the black dot represents the final border point which does not belong to any cluster. According to a certain cutoff distance, the maximum number of clusters is 3. If calculated in accordance with the empirical rule, the maximum number of clusters should be 14. Therefore, the algorithm can effectively reduce the iteration of FCM algorithm operation.

The selection principle of initial cluster centroids of density-based algorithm is that, usually, a cluster centroid is an object with higher local density, surrounded by neighbors with lower local density than it, and has a relatively large distance from other cluster centroids [4]. Density-based algorithm can automatically select the initial cluster centroids and determine the maximum number of clusters *c*_max⁡_ according to local density. The pseudocode is shown in Algorithm 1. These cluster centroids obtained are sorted in descending order according to local density.  Figure 2 demonstrates the process of density-based algorithm.

### 4.2. Fuzzy Clustering Validity Index

Firstly based on the geometric structure of the dataset, Xie and Beni put forward the Xie-Beni fuzzy clustering validity index [13], which tried to find a balance point between the fuzzy compactness and separation so as to acquire the optimal cluster result. The index is defined as

(5) $$\begin{array}{c} V_{\text{xie}}\left(\;U,V\;\right)=\frac{\left(1/n\right)\sum_{i=1}^{c}\sum_{j=1}^{n}u_{ij}^{m}{\left\|v_{i}-x_{j}\right\|}^{2}}{\min_{i\neq j}{\left\|v_{i}-v_{j}\right\|}^{2}}. \end{array}$$

In the formula, the numerator is the average distance from various objects to centroids, used to measure the compactness, and the denominator is the minimum distance between any two centroids, measuring the separation.

However, Bensaid et al. found that the size of each cluster had a large influence on Xie-Beni index and put forward a new index [14], which was insensitive to the number of objects in each cluster. Bensaid index is defined as

(6) $$\begin{array}{c} V_{B}\left(\;U,V\;\right)=\overset{c}{\underset{k=1}{\sum }}\frac{\sum_{i=1}^{n}u_{ik}^{m}{\left\|x_{i}-v_{k}\right\|}^{2}}{n_{k}\sum_{j=1}^{c}{\left\|v_{j}-v_{k}\right\|}^{2}}, \end{array}$$

where *n*_*k*_ is the fuzzy cardinality of the *k*th cluster and defined as

(7) $$\begin{array}{c} n_{k}=\overset{n}{\underset{i=1}{\sum }}u_{ik}. \end{array}$$

∑_*i*=1_^*n*^ *u*_*ik*_^*m*^‖*x*_*i*_ − *v*_*k*_‖^2^ shows the variation of the *k*th fuzzy cluster. Then, the compactness is computed as

(8) $$\begin{array}{c} \frac{1}{n_{k}}\overset{n}{\underset{i=1}{\sum }}u_{ik}^{m}{\left\|\;x_{i}-v_{k}\;\right\|}^{2}. \end{array}$$

∑_*j*=1_^*c*^‖*v*_*j*_ − *v*_*k*_‖^2^ denotes the separation of the *k*th fuzzy cluster, defined as the sum of the distances from its cluster centroid to the centroids of other *c* − 1 clusters.

Because

(9) $$\begin{array}{c} \underset{c\to n}{lim}{\left\|\;x_{i}-v_{k}\;\right\|}^{2}=0, \end{array}$$

this index is the same as the Xie-Beni index. When *c* → *n*, the index value will be monotonically decreased, close to 0, and will lose robustness and judgment function for determining the optimal number of clusters. Thus, this paper improves Bensaid index and proposes a new index *V*_*R*_:

(10) VRU,V=∑k=1c1/nk∑i=1nuikmxi−vk2+1/cvk−v−21/c−1∑j=1cvj−vk2.

The numerator represents the compactness of the *k*th cluster, where *n*_*k*_ is its fuzzy cardinality. Its second item, an introduced punishing function, denotes the distance from the cluster centroid of the *k*th cluster to the average of all cluster centroids, which can eliminate the monotonically decreasing tendency as the number of clusters increases to *n*. The denominator represents the mean distance from the *k*th cluster centroid to other cluster centroids, which is used for measuring the separation. The ratio of the numerator and the denominator thereof represents the clustering effect of the *k*th cluster. The clustering validity index is defined as the sum of the clustering effect (the ratio) of all clusters. Obviously, the smaller the value is, the better the clustering effect of the dataset is, and the corresponding *c* to the minimum value is the optimal number of clusters.

### 4.3. Self-Adaptive FCM

In this paper, the iterative trial-and-error process [16] is still used to determine the optimal number of clusters by self-adaptive FCM (SAFCM). The pseudocode of SAFCM algorithm is described in Algorithm 2.

## 5. Experiment and Analysis

**Figure 3:**
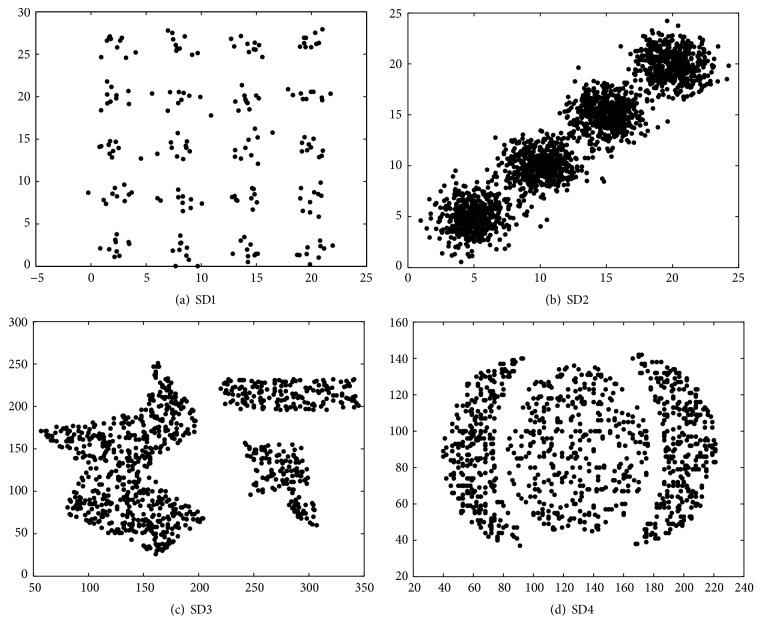
Four synthetic datasets.

This paper selects 8 experimental datasets, among which 3 datasets come from UCI datasets, respectively, Iris, Wine, and Seeds, a dataset (SubKDD) is randomly selected from KDD Cup 1999 shown in Table 1, and the remaining 4 datasets are synthetic datasets shown in Figure 3. SubKDD includes normal, 2 kinds of Probe attack (ipsweep and portsweep) and 3 kinds of DoS attack (neptune, smurf, and back). The first synthetic dataset (SD1) consists of 20 2-dimensional Gaussian distribution data with 10 samples. Their covariance matrixes are second-order unit matrix *I*_2_. The structural feature of the dataset is that the distance between any two clusters is large, and the number of classes is greater than $\left\lfloor \sqrt{n}\right\rfloor$. The second synthetic dataset (SD2) consists of 4 2-dimensional Gaussian distribution data. The cluster centroids are, respectively, (5,5), (10,10), (15,15), and (20,20), each containing 500 samples and the covariance matrix of each being 2*I*_2_. The structural feature of the dataset is of short intercluster distance with a small overlap. The third synthetic dataset (SD3) has a complicated structure. The fourth synthetic dataset (SD4) is a nonconvex one.

In this paper, the numeric value *x*_*ij*_ is normalized as

(11) $$\begin{array}{c} x_{ij}^{\prime }=\frac{x_{ij}-{\bar{x}}_{j}}{s_{j}}, \end{array}$$

where ${\bar{x}}_{j}$ and *s*_*j*_ denote the mean of the *j*th attribute value and its standard deviation, respectively; then

(12) x¯j=1n∑i=1nxij,sj=1n−1∑i=1nxij−x¯j2.

For the categorical attribute of SubKDD, the simple matching is used for the dissimilarity measure, that is, 0 for identical values and 1 for different values.

### 5.1. Experiment of *c*_max⁡_ Simulation

In [37], it was proposed that the number of clusters generated by AP algorithm could be selected as the maximum number of clusters. So this paper estimates the value of *c*_max⁡_ by the empirical rule, AP algorithm, and density-based algorithm. The specific experimental results are shown in Table 2. Let *p* = *p*_*m*_ in AP algorithm. *dc* is, respectively, selected as the distance of the first 1%, 2%, 3%, and 5%.

Experiment results show that, for the dataset with the true number of clusters greater than $\left\lfloor \sqrt{n}\right\rfloor$, it is obviously incorrect to let $c_{\max}=\lfloor \sqrt{n}\rfloor$. In other words, the empirical rule is invalid. The number of clusters finally obtained by AP algorithm is close to the actual number of clusters. For the dataset with the true number of clusters smaller than $\left\lfloor \sqrt{n}\right\rfloor$, the number of clusters generated by AP algorithm can be used as an appropriate *c*_max⁡_, but, sometimes, the value is greater than the value estimated by the empirical rule, which enlarges the search range of the optimal number of clusters. If *c*_max⁡_ is estimated by the proposed density-based algorithm, the results in most cases are appropriate. The method is invalid only on SD1 when the cutoff distance is the first 5% distance. When the cutoff distance is selected as the first 3% distance, *c*_max⁡_ generated by the proposed algorithm is much smaller than $\left\lfloor \sqrt{n}\right\rfloor$. It is closer to the true number of clusters and greatly narrows the search range of the optimal number of clusters. Therefore, the cutoff distance is selected as the distance of the first 3% in the later experiments.

### 5.2. Experiment of Influence of the Initial Cluster Centroids on the Convergence of FCM

To show that the initial cluster centroids obtained by density-based algorithm can quicken the convergence of clustering algorithm, the traditional FCM is adopted for verification. The number of clusters is assigned as the true value, with the convergence threshold being 10^−5^. Because the random selection of initial cluster centroids has a large influence on FCM, the experiment of each dataset is done repeatedly for 50 times, and the round numbers of the mean of algorithmic iterations are compared. The specific experimental result is shown in Table 3.

As shown in Table 3, the initial cluster centroids obtained by density-based algorithm can effectively reduce the iteration of FCM. Particularly, on SD1, the iteration of FCM is far smaller than that with randomly selected initial cluster centroids whose minimum iteration is 24 and maximum iteration reaches 63, with unstable clustering results. Therefore, the proposed method can not only effectively quicken the convergence of FCM, but also obtain a stable clustering result.

### 5.3. Experiment of Clustering Accuracy Based on Clustering Validity Index

For 3 UCI datasets and SubKDD, clustering accuracy *r* is adopted to measure the clustering effect, defined as

(13) $$\begin{array}{c} r=\frac{\sum_{i=1}^{k}b_{i}}{n}. \end{array}$$

Here *b*_*i*_ is the number of objects which co-occur in the *i*th cluster and the *i*th real cluster, and *n* is the number of objects in the dataset. According to this measurement, the higher the clustering accuracy is, the better the clustering result of FCM is. When *r* = 1, the clustering result of FCM is totally accurate.

In the experiments, the true number of clusters is assigned to each dataset and the initial cluster centroids are obtained by density-based algorithm. Then, experimental results of clustering accuracy are shown in Table 4.

Apparently, clustering accuracies of the datasets Wine, Seeds, and SubKDD are high, while that of the dataset Iris is relatively low for the reason that two clusters are nonlinearly separable.

**Figure 4:**
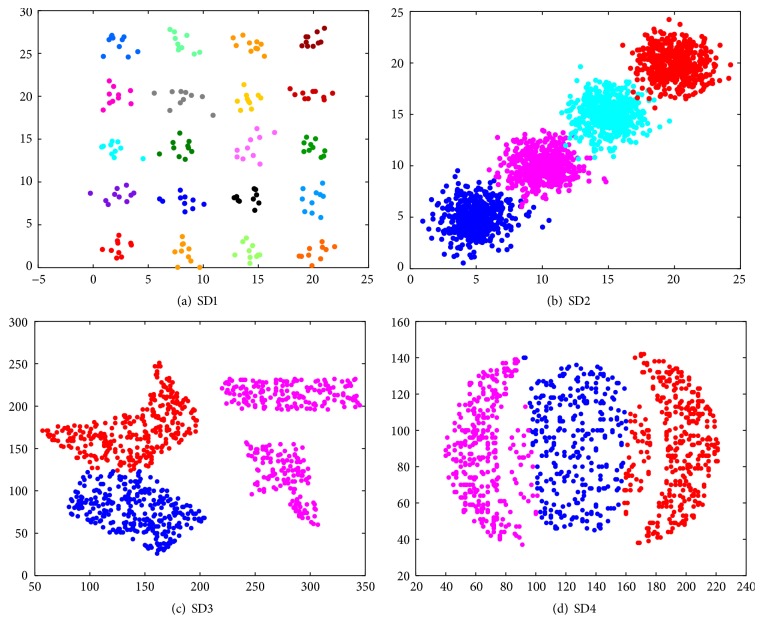
Clustering results of two synthetic datasets.

The clustering results of the proposed clustering validity index on 4 synthetic datasets are shown in Figure 4, which depict the good partitions on the datasets SD1 and SD2 and the slightly poor partition on SD3 and SD4 because of the complexity of their structure or nonconvexity. When the number of clusters is set as 4, SD3 is divided into 4 groups that means the right and the left each have 2 groups.

### 5.4. Experiment of the Optimal Number of Clusters

At last, Xie-Beni index, Bensaid index, Kwon index, and the proposed index are, respectively, adopted for running of SAFCM so as to determine the optimal number of clusters. The results are shown in Table 5. *V*_XB_, *V*_B_, *V*_K_, and *V*_R_ represent Xie-Beni index, Bensaid index, Kwon index, and the proposed index, respectively.

It shows that, for synthetic datasets SD1 and SD2 with simple structure, these indices can all obtain the optimal number of clusters. For 3 UCI datasets, SubKDD and SD4, Bensaid index cannot obtain an accurate number of clusters except SD3. For the dataset Wine, both Xie-Beni index and Kwon index can obtain the accurate number of clusters, while for the datasets Iris, Seeds, SubKDD, SD3, and SD4, they only obtain the result approximate to the true number of cluster. The proposed index obtains the right result on the datasets Wine and SD4 and a better result compared to Xie-Beni index and Kwon index on the datasets SubKDD and SD3, while it obtains the same result of the two indices on the datasets Iris and Seeds. There are two reasons. First, the importance degree of each property of the dataset is not considered in the clustering process but assumed to be the same, thereby affecting the experimental result. Second, SAFCM has a weak capacity to deal with overlapping clusters or nonconvexity dataset. These are the authors' subsequent research contents.

Tables 6 [Table tab7] [Table tab8] [Table tab9] [Table tab10] [Table tab11] [Table tab12]–13 list the detailed results of 4 indices on the experimental datasets in each iteration of SAFCM. For the dataset Wine, when the number of clusters is 4, 5, or 6, the result based on Xie-Beni index or Kwon index is of no convergence. However, the proposed index can achieve the desired results.

## 6. Conclusions

FCM is widely used in lots of fields. But it needs to preset the number of clusters and is greatly influenced by the initial cluster centroids. This paper studies a self-adaptive method for determining the number of clusters by using of FCM algorithm. In this method, a density-based algorithm is put forward at first, which can estimate the maximum number of clusters to reduce the search range of the optimal number, especially being fit for the dataset on which the empirical rule is inoperative. Besides, it can generate the high-quality initial cluster centroids so that the the clustering result is stable and the convergence of FCM is quick. Then, a new fuzzy clustering validity index was put forward based on fuzzy compactness and separation so that the clustering result is closer to global optimum. The index is robust and interpretable when the number of clusters tends to that of objects in the dataset. Finally, a self-adaptive FCM algorithm is proposed to determine the optimal number of clusters run in the iterative trial-and-error process.

The contributions are validated by experimental results. However, in most cases, each property plays a different role in the clustering process. In other words, the weight of properties is not the same. This issue will be focused on in the authors' future work.

## Figures and Tables

**Algorithm 1:**
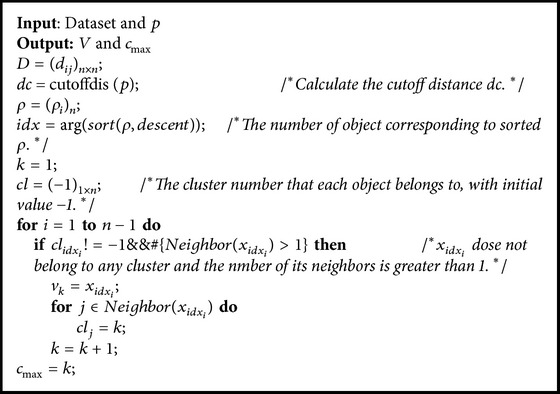
Density-based algorithm.

**Algorithm 2:**
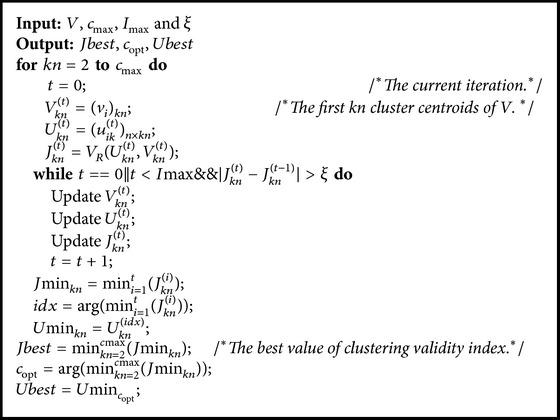
SAFCM.

**Table 1:** The data type and distribution of SubKDD.

Attack behavior	Number of samples
normal	200
ipsweep	50
portsweep	50
neptune	200
smurf	300
back	50

**Table 2:** *c*_max⁡_ estimated by several methods. *n* is the number of objects in the dataset, *c* is the actual number of clusters, *c*_ER_ is the number of clusters estimated by the empirical rule, that is, $\left\lfloor \sqrt{n}\right\rfloor$, *c*_AP_ is the number of clusters obtained by AP algorithm, and *c*_DBA_ is the number of clusters obtained by density-based algorithm.

					*c*_DBA_			
Dataset	*n*	*c*	*c*_ER_	*c*_AP_	*p* = 1	*p* = 2	*p* = 3	*p* = 5
Iris	150	3	12	9	20	14	9	6
Wine	178	3	13	15	14	7	6	3
Seeds	210	3	14	13	18	12	5	2
SubKDD	1050	6	32	24	21	17	10	7
SD1	200	20	14	19	38	22	20	—
SD2	2000	4	44	25	16	3	4	2
SD3	885	3	29	27	24	19	5	3
SD4	947	3	30	31	23	13	8	4

**Table 3:** Comparison of iterations of FCM algorithm. Method 1 uses the random initial cluster centroids, and Method 2 uses the cluster centroids obtained by density-based algorithm.

Dataset	Method 1	Method 2
Iris	21	16
Wine	27	18
Seeds	19	16
SubKDD	31	23
SD1	38	14
SD2	18	12
SD3	30	22
SD4	26	21

**Table 4:** Clustering accuracy.

Dataset	Iris	Wine	Seeds	SubKDD
Clustering accuracy	84.00%	96.63%	91.90%	94.35%

**Table 5:** Optimal number of clusters estimated by several clustering validity indices.

Dataset	*V*_XB_	*V*_B_	*V*_K_	*V*_R_
Iris	2	9	2	2
Wine	3	6	3	3
Seeds	2	5	2	2
SubKDD	10	10	9	4
SD1	20	20	20	20
SD2	4	4	4	4
SD3	5	3	5	4
SD4	2	8	2	3

**Table 6:** The value of clustering validity index on Iris.

*c*	*V*_XB_	*V*_B_	*V*_K_	*V*_R_
2	**0.114234**	0.223604	**17.384973**	**0.473604**
3	0.223973	0.124598	34.572146	0.551565
4	0.316742	0.099103	49.279488	0.615436
5	0.560109	0.089108	87.540968	0.676350
6	0.574475	0.072201	90.563379	0.691340
7	0.400071	0.067005	63.328679	0.735311
8	0.275682	0.036283	45.736972	0.614055
9	0.250971	**0.027735**	42.868449	0.584244

**Table 7 tab7:** The value of clustering validity index on Wine.

*c*	*V*_XB_	*V*_B_	*V*_K_	*V*_R_
2	0.663406	1.328291	118.33902	1.578291
3	**0.468902**	0.513071	**83.939058**	**1.346421**
4	—	0.473254	—	1.735791
5	—	0.373668	—	1.846686
6	—	**0.256698**	—	1.683222

**Table 8 tab8:** The value of clustering validity index on Seeds.

*c*	*V*_XB_	*V*_B_	*V*_K_	*V*_R_
2	**0.147247**	0.293609	**31.170349**	**0.543609**
3	0.212127	0.150899	45.326216	0.599001
4	0.243483	0.127720	52.215334	0.697943
5	0.348842	**0.085477**	75.493654	0.701153

**Table 9 tab9:** The value of clustering validity index on SubKDD.

*c*	*V*_XB_	*V*_B_	*V*_K_	*V*_R_
2	0.646989	1.324434	550.166676	1.574431
3	0.260755	0.378775	222.020838	1.090289
4	0.133843	0.062126	119.544560	**0.511238**
5	0.234402	0.052499	202.641204	0.537852
6	0.180728	0.054938	156.812271	0.583800
7	0.134636	0.047514	119.029265	0.619720
8	0.104511	0.032849	91.9852740	0.690873
9	0.129721	0.027639	**74.0128470**	0.562636
10	**0.06822**	**0.027025**	91.3528560	0.528528

**Table 10 tab10:** The value of clustering validity index on SD1.

*c*	*V*_XB_	*V*_B_	*V*_K_	*V*_R_
2	0.221693	0.443390	44.592968	0.693390
3	0.206035	0.198853	40.264251	0.726245
4	0.127731	0.093653	26.220200	0.655550
5	0.130781	0.069848	27.154867	0.651465
6	0.144894	0.050067	22.922121	0.639325
7	0.136562	0.040275	29.126152	0.636258
8	0.112480	0.032874	24.323625	0.627442
9	0.115090	0.026833	24.242580	0.624936
10	0.141415	0.022611	28.574579	0.616701
11	0.126680	0.019256	28.821707	0.611524
12	0.103178	0.016634	23.931865	0.605990
13	0.110355	0.013253	26.517065	0.588246
14	0.095513	0.011083	23.635022	0.576808
15	0.075928	0.009817	19.302095	0.562289
16	0.066025	0.008824	17.236138	0.557990
17	0.054314	0.007248	14.995284	0.544341
18	0.045398	0.006090	13.208810	0.534882
19	0.039492	0.005365	11.977437	0.527131
20	**0.034598**	**0.004917**	**10.835952**	**0.517481**

**Table 11 tab11:** The value of clustering validity index on SD2.

*c*	*V*_XB_	*V*_B_	*V*_K_	*V*_R_
2	0.066286	0.132572	132.81503	0.382572
3	0.068242	0.063751	137.52535	0.394200
4	**0.060916**	**0.028149**	**123.09967**	**0.378337**

**Table 12 tab12:** The value of clustering validity index on SD3.

*c*	*V*_XB_	*V*_B_	*V*_K_	*V*_R_
2	0.148379	0.300899	131.557269	0.570876
3	0.195663	**0.142149**	173.900551	0.599680
4	0.127512	0.142150	113.748947	**0.557198**
5	**0.126561**	0.738070	**113.262975**	0.589535

**Table 13:** The value of clustering validity index on SD4.

*c*	*V*_XB_	*V*_B_	*V*_K_	*V*_R_
2	**0.104434**	0.208832	**99.1482710**	0.473748
3	0.170326	0.142561	162.044450	**0.458832**
4	0.221884	0.081007	211.692699	0.583529
5	0.156253	0.053094	157.683921	0.603211
6	0.123191	0.041799	118.279116	0.575396
7	0.165465	0.032411	107.210082	0.592625
8	0.145164	**0.028698**	139.310969	0.606049
